# Association Between Problematic TikTok Use and Procrastination, Loneliness, and Self-Esteem: A Moderation Analysis by Sex and Generation

**DOI:** 10.3390/ejihpe15100209

**Published:** 2025-10-13

**Authors:** Aglaia Katsiroumpa, Zoe Katsiroumpa, Evmorfia Koukia, Polyxeni Mangoulia, Parisis Gallos, Ioannis Moisoglou, Petros Galanis

**Affiliations:** 1 Clinical Epidemiology Laboratory, Faculty of Nursing, National and Kapodistrian University of Athens, 11527 Athens, Greece; zkatsiroumpa@nurs.uoa.gr (Z.K.); pegalan@nurs.uoa.gr (P.G.); 2 Laboratory Nursing Counselling and Psychoeducation of Patients and Caregivers, Faculty of Nursing, National and Kapodistrian University of Athens, 11527 Athens, Greece; ekoukia@nurs.uoa.gr (E.K.); pmango@nurs.uoa.gr (P.M.); 3 Faculty of Nursing, University of West Attica, 12243 Athens, Greece; parisgallos@nurs.uoa.gr; 4 Faculty of Nursing, University of Thessaly, 41500 Larissa, Greece; iomoysoglou@uth.gr

**Keywords:** TikTok, procrastination, loneliness, self-esteem, TikTok Addiction Scale, sex, age

## Abstract

The aim of this study was to examine the association between problematic TikTok use and procrastination, loneliness, and self-esteem in Greece. Moreover, we performed a moderation analysis to examine potential moderators such as sex and age. We conducted a cross-sectional study with a convenience sample of 1033 TikTok users. We used the TikTok Addiction Scale to measure problematic TikTok use. Additionally, we measured procrastination, loneliness, and self-esteem with the Irrational Procrastination Scale, the UCLA 3-Item Loneliness Scale, and the Rosenberg Self-Esteem Scale, respectively. We performed moderation analysis using linear regression models. We found a positive association between problematic TikTok use, procrastination, and loneliness. Also, we found a negative association between problematic TikTok use and self-esteem. Moderation analysis showed a more prominent association between problematic TikTok use and procrastination among females and Generation Z. The association between problematic TikTok use and loneliness was stronger among males and Generation X. The association between problematic TikTok use and self-esteem was stronger among males and Generation Z. In conclusion, our study supports the negative effect of problematic TikTok use on users. Moreover, sex and gender are moderators in these associations. However, due to study limitations, further research should be conducted.

## 1. Introduction

A widely known trait of our times refers to the domination of social media, which spread to high numbers, becoming part of daily life for most of the world, especially for young people (Statista, 2025). Using extremely updated and smart algorithms, social media can easily and in detail “read” their users, figure out their personality, and introduce to them contents of high relevancy to their taste and interest (Kim, 2017; Swart, 2021). According to the statistics, there were 5.44 billion users in 2025, which are expected to rise to 6.46 by the end of 2029 (Statista, 2025).

Among all social media platforms, TikTok is the most prominent one. With the new generations spending most of their day watching videos on TikTok, it is characterized as the most powerful social media platform (Katsiroumpa et al., 2025c; Statista, 2025). TikTok gathers 1.5 billion users monthly, with 35% of its users referring to Generation Z (age group of 16 to 24 years old) (Katsiroumpa et al., 2025c; Statista, 2025). Both statistics and the literature claim that TikTok entails a great load of addiction (Koetsier, 2024; Montag et al., 2021; Pedrouzo & Krynski, 2023). According to United States statistics, there were almost common rates among generations regarding addiction and negative mental health association. More specifically, Generation Z comes first, with 77.7% of them supporting that TikTok seems to be addictive, followed by the Millennials (73.5%) and Gen X (71.7%) (Statista, 2023).

Although the up to 60 s videos that TikTok produce are usually used for casual, harmless reasons (such as informing, promotion, and updates concerning trends) and they can be a potential creative environment for self-expression, the literature claims several concerning issues. First, it is estimated that the time users devote to TikTok may span from 52 to 150 min or more (Statista, 2025; Oberlo, 2023). This fact, along with the psychological effect TikTok reflects on users, raises addiction-related concerns. TikTok follows the exact same pathway as any other addictive habit: the dopaminergic system (Andreassen et al., 2016; Campanella, 2024; Chung, 2018). In fact, TikTok has a double way to allure and conquer its users, first, by providing joy and pleasure to the TikTokers via recognition and, secondly, with the euphoria it brings by the constant consumption of videos highly relevant to users’ interests and tastes. One way or another, TikTok engages its users, since both ways lead to the stimulation of the dopaminergic system and the reward mechanism, which is responsible for obtaining pleasure (Koetsier, 2024; Montag et al., 2021). As the individuals try to re-experience the initial pleasure by repeating it, they end up addicted, craving for more (Caponnetto et al., 2025; Koetsier, 2024; Montag et al., 2021). It was indicated that, as per 2023 statistics in a United States survey, participants attributed addictive behaviors towards TikTok and effects on mental health. More specifically, 73.5% of the participants agreed that TikTok is addictive, while 26.8% blamed the platform for negative mental health as a result of TikTok usage (Statista, 2023).

The impact of TikTok on individuals’ self-esteem is also very questionable. The term of self-esteem refers to the degree of positive perception of one’s image. According to the American Psychological Association “self-esteem reflects a person’s physical self-image, view of their accomplishments and capabilities, and values and perceived success in living up to them, as well as the ways in which others view and respond to that person” (American Psychological Association, 2023). Two meta-analyses showed that self-esteem increases as individuals get older. In addition, men tend to have stronger self-esteem than women (Bleidorn et al., 2016; Kling et al., 1999). Concerning TikTok and its relationship to self-esteem, the literature indicates that the impact of unrealistic models and lifestyles that TikTok promotes ends up with self-esteem-related problems for users (Bissonette Mink & Szymanski, 2022; Conte et al., 2025; Galanis et al., 2024a). Studies suggest that women seem to be more burdened with self-esteem issues because of the constant display of ideal, non-realistic standards concerning their image. Even TikTok campaigns regarding the enhancement of self-esteem fail their purpose, ending in worse results concerning self-perception and self-esteem (Bissonette Mink & Szymanski, 2022; Harriger et al., 2023; Ibn Auf et al., 2023; Seekis & Kennedy, 2023).

Another important question is how TikTok isolates its users. It derives from several studies that individuals, and most frequently boys, are feeling uncomfortable and isolated when they are not online and away from social media (including TikTok). More specifically, they declare lack of communication and a feeling of isolation (Conte et al., 2025; Muñoz-Rodríguez et al., 2023; Savoia et al., 2021; Sipal et al., 2011). This finding can be explained in the context of “fear of missing out” phenomenon, where social media and internet users feel that they are missing important information and social updates when they go offline (Bao & Li, 2025; X. Li & Ye, 2022; Wu et al., 2025). These findings show that real-world connection and interpersonal touch seem to be understudied by the digital companion. In addition to the above, the literature supports that loneliness is increased among TikTok users. Either due to total absence of real-world personal relationships or due to poor quality in interpersonal connection, users tend to feel more lonely (Chao et al., 2023; Harries et al., 2025; Huang, 2017; Smith & Short, 2022). Although, studies from the general population show that, as people, especially women, get older, they experience more loneliness (Nicolaisen & Thorsen, 2024; Pagan, 2020; Wang et al., 2023), according to Pop et al., things are reversed, meaning that the younger the users of social media are, the more lonely they feel (Pop et al., 2022).

Studies have supported that social media and mobile phone addiction along with the “fear of missing out” phenomenon have been linked to procrastination especially among young people (Bao & Li, 2025; X. Li & Ye, 2022; Wu et al., 2025). This means that individuals are pushing their responsibilities back in order to spend more time on social media platforms, thinking that, if they do so, they will stay connected to other users and updated (Fuentes Chavez et al., 2025; X. Li & Ye, 2022; Naushad et al., 2025; Zhou et al., 2024). Doomscrolling—the compulsive consumption of negative news on digital platforms—has emerged as a significant contributor to procrastination. Research indicates that individuals often turn to doomscrolling as an avoidance strategy when faced with tasks that evoke stress, boredom, or fear of failure. This behavior provides immediate gratification through novelty and emotional engagement, while the tasks being avoided typically offer delayed rewards. Consequently, doomscrolling not only consumes valuable time but also heightens anxiety and cognitive overload, further impairing focus and productivity. Studies have shown a strong correlation between doomscrolling and procrastination, suggesting that this habit perpetuates a self-reinforcing cycle of avoidance and diminished well-being (Punzalan et al., 2024; Satici et al., 2023; I. M. Hughes et al., 2024). We should mention that TikTok and YouTube Shorts serve similar purposes—short-form video sharing—but their core functions differ significantly. TikTok operates as a standalone platform built entirely around short, trend-driven videos, prioritizing entertainment and rapid content discovery through its highly personalized “For You Page”. Its design encourages viral growth and interactive engagement. In contrast, YouTube Shorts is integrated into the broader YouTube ecosystem, functioning as an entry point to long-form content and channel growth. While TikTok focuses on quick, ephemeral trends, YouTube Shorts emphasizes sustained creator development, leveraging searchability, watch time, and monetization. Essentially, TikTok functions as a viral content engine, whereas YouTube Shorts acts as a strategic tool for long-term audience building and monetization. Smartphones and social media have been indicated as responsible not only for “bed-time” but also academic procrastination among students, especially among male students (Naushad et al., 2025). According to studies, self-control seems to play a key role to the limitation of procrastination (Geng et al., 2021; Rasouli et al., 2025). Lastly, smartphone addiction has been correlated to poor mental health such as depression and anxiety due to bed-time procrastination (Geng et al., 2021). Procrastination is a major factor since it is responsible for poor academic and school performance, especially among boys who tend to skip their homework more often than girls (Chao et al., 2023; Rasouli et al., 2025; Sipal et al., 2011; Zacks & Hen, 2018). Furthermore, procrastination has been associated with poor sleep and bad quality of night-time sleep (Hill et al., 2022; Ma et al., 2022).

In brief, research has shown that social media and mobile phone addiction, combined with the fear of missing out, are strongly associated with procrastination, particularly among young people (Bao & Li, 2025; X. Li & Ye, 2022; Wu et al., 2025). However, no studies until now have investigated the association between problematic TikTok use and procrastination. Additionally, the literature regarding the association between problematic TikTok use and self-esteem is limited since only three studies have identified a negative association between these variables (Bissonette Mink & Szymanski, 2022; Conte et al., 2025; Galanis et al., 2024a). In particular, the promotion of unrealistic models and lifestyles on TikTok often leads to self-esteem issues among its users, especially among females. Moreover, limited research indicates that TikTok users experience higher levels of loneliness, often due to a complete lack of real-world personal relationships or the poor quality of their interpersonal connections (Chao et al., 2023; Harries et al., 2025; Huang, 2017; Smith & Short, 2022). In this context, we investigated the association between problematic TikTok use and procrastination, loneliness, and self-esteem. Moreover, we examined the role of sex and age as potential modifiers. In detail, the research hypotheses of this study were the following:

**H1.**  *Problematic TikTok use would be positively associated with procrastination.*

**H2.**  *Sex would moderate the association between problematic TikTok use and procrastination.*

**H3.**  *Age would moderate the association between problematic TikTok use and procrastination.*

**H4.**  *Problematic TikTok use would be positively associated with loneliness.*

**H5.**  *Sex would moderate the association between problematic TikTok use and loneliness.*

**H6.**  *Age would moderate the association between problematic TikTok use and loneliness.*

**H7.**  *Problematic TikTok use would be negatively associated with self-esteem.*

**H8.**  *Sex would moderate the association between problematic TikTok use and self-esteem.*

**H9.**  *Age would moderate the association between problematic TikTok use and self-esteem.*

## 2. Materials and Methods

### 2.1. Study Design

We conducted a web-based cross-sectional study in Greece, utilizing an online version of the study questionnaire developed through Google Forms, which was disseminated via TikTok. Data collection occurred from January to March 2025. Specifically, we produced a TikTok video to inform users about our study. Participants were required to be adults over the age of 18. The Google Forms link was sent to interested TikTok users through inbox messages. Prior to commencing the online survey, participants were presented with an introductory page containing essential information. This page detailed the study’s purpose and design, provided a brief overview of the questions, estimated the time required to complete the questionnaire, emphasized the voluntary nature of participation, and informed participants of their ability to exit the survey by closing their web browser. Additionally, our contact information was provided to the participants. To ensure data integrity, we inquired whether participants had previously completed the survey, and any affirmative responses were subsequently excluded from the dataset, resulting in a convenience sample. We applied the Reporting of Observational Studies in Epidemiology (STROBE) (Vandenbroucke et al., 2007) to perform our study.

We used G*Power v.3.1.9.2 (Düsseldorf, Germany) to calculate our sample size. Considering a small effect size between problematic TikTok use and procrastination, loneliness, and self-esteem (Cohen’s f^2^ = 0.02), the number of independent variables (one predictor and seven confounders), a confidence level of 99%, and a margin error of 5%, sample size was estimated at 921 participants. Given that the final sample in this study comprised 1033 participants and the calculated effect sizes, the post hoc power was estimated at 99.5%.

### 2.2. Measurements

We assessed problematic TikTok use using the TikTok Addiction Scale (TTAS), which consists of 15 items and evaluates six dimensions: salience (two items), mood modification (two items), tolerance (three items), withdrawal symptoms (two items), conflict (four items), and relapse (two items) (Galanis et al., 2024c). Salience pertains to users’ preoccupation with TikTok, while mood modification refers to its ability to improve emotional well-being. Tolerance is evident when users need to engage more with TikTok to feel satisfied, and withdrawal is characterized by negative feelings when they stop using it. Conflict occurs when TikTok disrupts daily life, while relapse is when users revert to previous usage patterns after abstaining. The TTAS evaluates individuals’ attitudes towards TikTok over the past year, with responses on a five-point Likert scale from 1 (very rarely) to 5 (very often). Scores for the TTAS and the six factors range from 1 to 5, with higher scores indicating more problematic TikTok use. The response range for the six subscales ranges from 1 to 5. Also, the response range for the entire scale ranges from 1 to 5. Total score for the entire scale and the subscales is calculated as a mean score of all answers on items. A cut-off score of 3.23 is suggested to differentiate between healthy and problematic users (Galanis et al., 2024b). This cut-off score refers to the mean score of the total scale. The variable that we analyzed is degree of addiction to TikTok (response range from 1 to 5). We used the valid Greek version of the TTAS (Katsiroumpa et al., 2025c). In our study, the TTAS had a Cronbach’s alpha of 0.944, and the six factors had Cronbach’s alphas ranging from 0.689 to 0.911. Comparative fit index (CFI), Tucker–Lewis index (TLI), and root mean square error of approximation (RMSEA) for the TTAS were 0.984, 0.982, and 0.043, respectively.

We measured procrastination with the Irrational Procrastination Scale (IPS) (Steel, 2010). The IPS includes nine items, such as “I delay tasks beyond what is reasonable” and “I do everything when I believe it needs to be done”. Answers are on a five-point Likert scale from 1 (very seldom or not true of me) to 5 (very often true, or true of me). Total score on the IPS ranges from 9 to 45, with higher scores indicating higher levels of procrastination. The variable that we analyzed is degree of procrastination (response range from 9 to 45). We used the valid Greek version of the IPS (Katsiroumpa et al., 2025a). In our study, the TTAS had a Cronbach’s alpha of 0.893. CFI, TLI, and RMSEA for the IPS were 0.958, 0.944, and 0.077, respectively.

We measured loneliness with the UCLA 3-Item Loneliness Scale (UCLA-LS-3) (M. E. Hughes et al., 2004). The UCLA-LS-3 includes three items, such as “How often do you feel that you lack companionship?” and “How often do you feel left out?” Answers are on a three-point Likert scale from 1 (hardly ever) to 3 (often). Total score on the UCLA-LS-3 ranges from 3 to 9, with higher scores indicating higher levels of loneliness. The variable that we analyzed is degree of loneliness (response range from 3 to 9). We used the valid Greek version of the UCLA-LS-3 (Katsiroumpa et al., 2025b). In our study, the UCLA-LS-3 had a Cronbach’s alpha of 0.807. CFI, TLI, and RMSEA for the UCLA-LS-3 were 0.988, 0.993, and 0.001, respectively.

We measured self-esteem with the Rosenberg Self-Esteem Scale (RSES) (Rosenberg, 1965). The RSES includes 10 items, such as “On the whole, I am satisfied with myself” and “I feel that I have a number of good qualities”. Answers are on a four-point Likert scale from 1 (strongly disagree) to 4 (strongly agree). Total score on the RSES ranges from 10 to 40, with higher scores indicating higher levels of self-esteem. The variable that we analyzed is level of self-esteem (response range from 10 to 40). We used the valid Greek version of the RSES (Galanou et al., 2014). In our study, the RSES had a Cronbach’s alpha of 0.888. CFI, TLI, and RMSEA for the RSES were 0.995, 0.982, and 0.071, respectively.

We measured the following demographic variables: sex (females or males), age (continuous variable), educational level (elementary school, high school, university degree, MSc diploma, or PhD diploma), socioeconomic status, TikTok use per day (continuous variable), social media use per day (continuous variable), and total number of social media accounts (continuous variable). We categorized our participants into three age generations. In particular, Generation Z referred to people born between 1997 and 2012, Millennials referred to people born between 1981 and 1996, and Generation X referred to people born between 1965 and 1980 (Dimock, 2019). We measured socioeconomic status with a simple question: “How do you consider your socioeconomic status?” Answers were on a scale from 0 to 10, where 0 referred to the worst socioeconomic status and 10 to the best socioeconomic status.

### 2.3. Participants

Our study population included 1033 participants. Table 1 shows demographic characteristics of our participants. In our sample, 75.4% were females, while 24.6 were males. Mean age was 31.1 years (SD; 12.4), with a median of 26.0 years. Most participants belonged to Generation Z (53.6%), while 28.6% belonged to Millennials and 17.8% belonged to Generation X. Among our participants, 60.4% possessed a university degree, while 39.6% attended high school. The mean value on the socioeconomic status scale denoted a moderate level.

### 2.4. Ethical Issues

We conducted our study according to the guidelines of the Declaration of Helsinki (World Medical Association, 2013). Our study protocol was approved by the Ethics Committee of the Faculty of Nursing, National and Kapodistrian University of Athens (approval number; 05, 10 October 2024). Participants were informed about the study design and asked to consent to participate. Specifically, before accessing the online questionnaire, TikTok users were asked via Google Forms if they agreed to participate. Those who consented were allowed to complete the questionnaire, thereby obtaining informed consent. Furthermore, no identifying data were collected from participants, ensuring that participation was both voluntary and anonymous.

### 2.5. Statistical Analysis

We present categorical variables as numbers (n) and percentages (%) and continuous variables with mean, standard deviation (SD), median, and interquartile range. We used the Kolmogorov–Smirnov test and Q-Q plots to assess the distribution of continuous variables. We considered TikTok addiction as the independent variable. We found moderate to high correlations between the six factors on the TTAS since correlation coefficients ranged from 0.554 to 0.771 (*p*-value < 0.001 in all cases). Thus, to avoid multicollinearity in the multivariable regression models, we chose to use the total score on the TTAS as the independent variable. Our dependent variables were procrastination, loneliness, and self-esteem scores. Demographic and social media characteristics were considered as potential confounders. We performed moderation analysis (Hair et al., 2021) to examine if sex and age are moderators in the association between problematic TikTok use and procrastination, loneliness, and self-esteem. We centered the independent variable (TTAS score) and the moderator variable (age) to reduce multicollinearity (Iacobucci et al., 2017). For instance, “centered” variable of TTAS score was obtained by subtracting the mean TTAS score from each individual TTAS score value. Similarly, we calculated the “centered” variable of age. Since the dependent variables were continuous and normally distributed, we applied linear regression analysis, presenting unstandardized and standardized coefficients beta, 95% confidence intervals (CI), and *p*-values. First, we created a linear regression model including the independent variable (TTAS score) and the moderator (sex or age). Then, we created a second regression by adding the interaction term (TTAS score ∗ sex or age). We calculated the total variance (R^2^) of the dependent variable explained in the two models. Statistically significant coefficients beta for the interaction term indicated the presence of moderation. Moreover, we performed simple slope analysis to identify the direction of the moderating effects introduced by sex and age. *p*-values less than 0.05 were considered statistically significant. We used the IBM SPSS 28.0 (IBM Corp. Released 2021. IBM SPSS Statistics for Windows, Version 28.0. Armonk, NY, USA, IBM Corp.) for analysis.

## 3. Results

### 3.1. Social Media Characteristics

Mean TikTok use per day was 1.8 h (SD, 1.5), with a median of 1 hour, a minimum value of 30 min, and a maximum value of 8.0 h. Mean TikTok use per day was 1.8 h (SD, 1.5) for females and 1.7 h (SD, 1.5) for males. Mean TikTok use per day was 2.3 h (SD, 1.7) for Generation Z, 1.3 h (SD, 1.1) for Millennials, and 0.8 h (SD, 0.5) for Generation X.

The mean social media use per day was 3.3 h (SD, 1.9), with a median of 3.0 h, a minimum value of 30 min, and a maximum value of 8.0 h. Mean social media use per day was 3.4 h (SD, 1.9) for females and 3.3 h (SD, 1.8) for males. Mean social media use per day was 4.0 h (SD, 1.8) for Generation Z, 2.9 h (SD, 1.7) for Millennials, and 2.1 h (SD, 1.4) for Generation X.

Most participants (92.9%, n = 960) had accounts on at least two social media platforms. In particular, 7.1% (n = 73) had an account only on TikTok, 15.8% (n = 163) had accounts on two social media platforms, 28.0% (n = 289) on three social media platforms, 21.9% (n = 226) on four social media platforms, 15.4% (n = 159) on five social media platforms, and 11.9% (n = 123) on 6–8 social media platforms. Mean number of accounts was 3.6 (SD; 1.5), with a median of 3.0, a minimum value of 1, and a maximum value of 8.

### 3.2. Association Between Problematic TikTok Use and Procrastination with Sex as the Moderator

**Figure 1:**
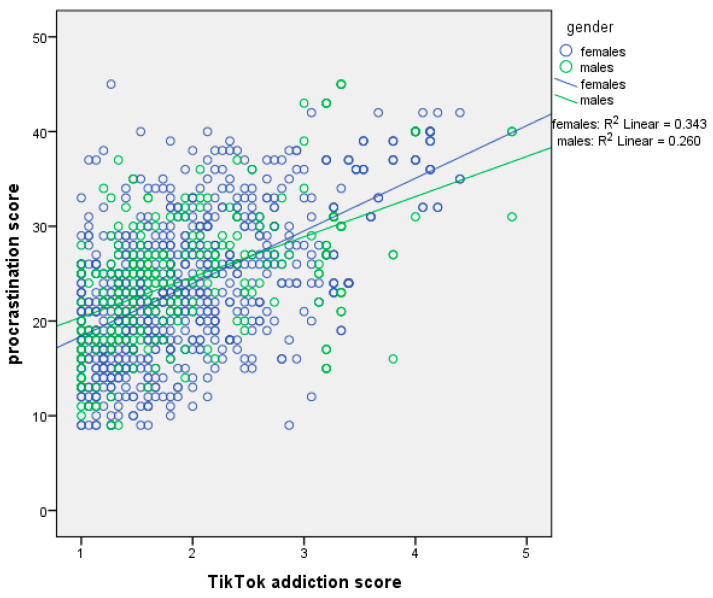
Simple slopes analysis with procrastination score as the dependent variable and sex as the moderator.

Table 2 presents moderation analysis with procrastination score as the dependent variable and sex as the moderator. The first regression model, which included the independent variable (centered TikTok addiction score) and the moderator (sex), explained 32.1% of the variance in procrastination score (R^2^ = 32.1%, *p*-value < 0.001). The second regression model, which added the interaction term (TikTok addiction score ∗ sex), explained an additional 0.4% of the variance (ΔR^2^ = 0.4%, *p*-value = 0.013), bringing the total variance explained to 32.5% (R^2^ = 32.5%, *p*-value < 0.001). In the first model, TikTok addiction score had a significant effect on procrastination. We found a positive association between TikTok addiction score and procrastination. In the second model, both TikTok addiction score and sex remained significant, and the interaction term also significantly predicted procrastination. Thus, sex moderated the association between TikTok addiction score and procrastination. Simple slopes analysis indicated a stronger association between TikTok addiction score and procrastination among females (Figure 1).

### 3.3. Association Between Problematic TikTok Use and Procrastination with Age as the Moderator

**Figure 2:**
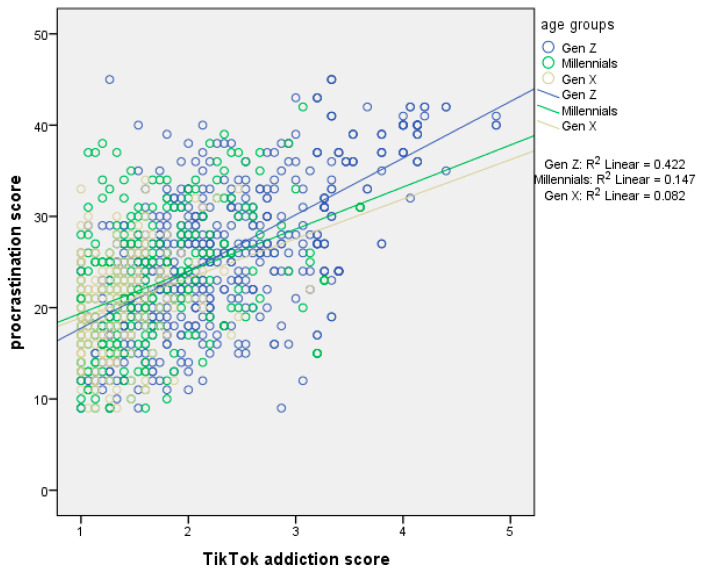
Simple slopes analysis with procrastination score as the dependent variable and age as the moderator.

Table 3 presents moderation analysis with procrastination score as the dependent variable and age as the moderator. The first regression model, which included the independent variable (centered TikTok addiction score) and the moderator (age), explained 36.2% of the variance in procrastination score (R^2^ = 36.2%, *p*-value < 0.001). The second regression model, which added the interaction term (TikTok addiction score ∗ age), explained an additional 0.6% of the variance (ΔR^2^ = 0.4%, *p*-value = 0.011), bringing the total variance explained to 36.8% (R^2^ = 36.8%, *p*-value < 0.001). In the first model, TikTok addiction score had a significant effect on procrastination. We found a positive association between TikTok addiction score and procrastination. In the second model, both TikTok addiction score and age remained significant, and the interaction term also significantly predicted procrastination. Thus, age moderated the association between TikTok addiction score and procrastination. Simple slopes analysis indicated a stronger association between TikTok addiction score and procrastination in Generation Z (Figure 2).

### 3.4. Association Between Problematic TikTok Use and Loneliness with Sex as the Moderator 

**Figure 3:**
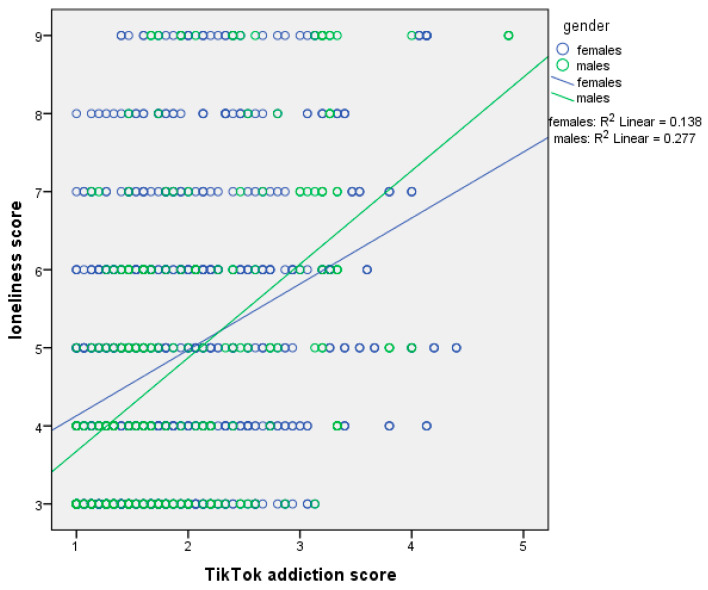
Simple slopes analysis with loneliness score as the dependent variable and sex as the moderator.

Table 4 presents moderation analysis with loneliness score as the dependent variable and sex as the moderator. The first regression model, which included the independent variable (centered TikTok addiction score) and the moderator (sex), explained 16.9% of the variance in loneliness score (R^2^ = 16.9%, *p*-value < 0.001). The second regression model, which added the interaction term (TikTok addiction score ∗ sex), explained an additional 0.4% of the variance (ΔR^2^ = 0.4%, *p*-value = 0.015), bringing the total variance explained to 17.3% (R^2^ = 17.3%, *p*-value < 0.001). In the first model, TikTok addiction score had a significant effect on loneliness. We found a positive association between TikTok addiction score and loneliness. In the second model, both TikTok addiction score and sex remained significant, and the interaction term also significantly predicted loneliness. Thus, sex moderated the association between TikTok addiction score and loneliness. Simple slopes analysis indicated a stronger association between TikTok addiction score and loneliness among males (Figure 3).

### 3.5. Association Between Problematic TikTok Use and Loneliness with Age as the Moderator

**Figure 4:**
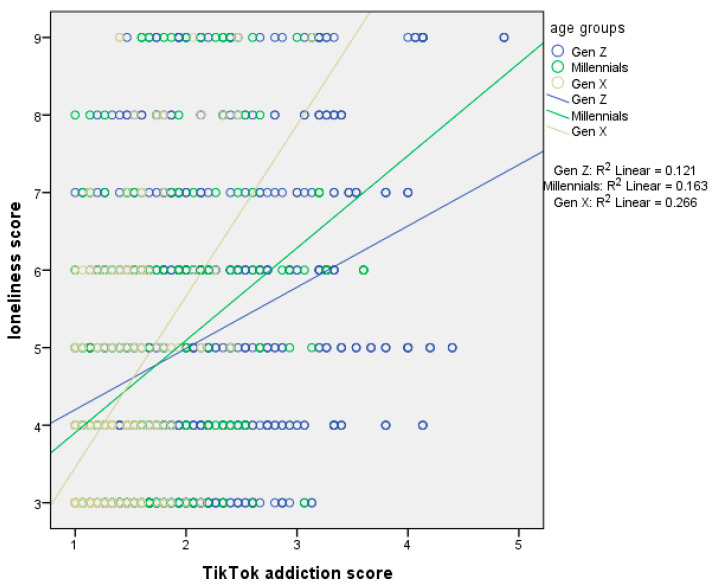
Simple slopes analysis with loneliness score as the dependent variable and age as the moderator.

Table 5 presents moderation analysis with loneliness score as the dependent variable and age as the moderator. The first regression model, which included the independent variable (centered TikTok addiction score) and the moderator (age), explained 16.9% of the variance in loneliness score (R^2^ = 16.9%, *p*-value < 0.001). The second regression model, which added the interaction term (TikTok addiction score ∗ age), explained an additional 1.3% of the variance (ΔR^2^ = 1.3%, *p*-value < 0.001), bringing the total variance explained to 18.2% (R^2^ = 18.2%, *p*-value < 0.001). In the first model, TikTok addiction score had a significant effect on loneliness. We found a positive association between TikTok addiction score and loneliness. In the second model, both TikTok addiction score and age remained significant, and the interaction term also significantly predicted loneliness. Thus, age moderated the association between TikTok addiction score and loneliness. Simple slopes analysis indicated a stronger association between TikTok addiction score and procrastination in Generation Χ (Figure 4).

### 3.6. Association Between Problematic TikTok Use and Self-Esteem with Sex as the Moderator 

**Figure 5:**
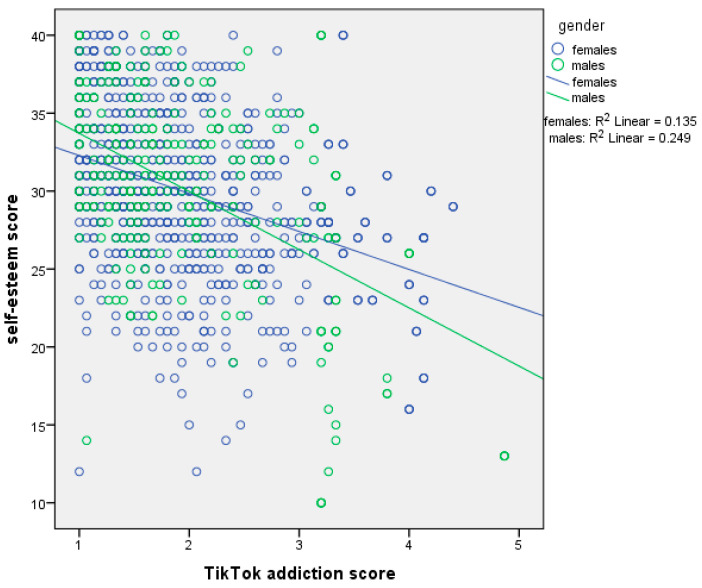
Simple slopes analysis with self-esteem score as the dependent variable and sex as the moderator.

Table 6 presents moderation analysis with self-esteem score as the dependent variable and sex as the moderator. The first regression model, which included the independent variable (centered TikTok addiction score) and the moderator (sex), explained 16.4% of the variance in self-esteem score (R^2^ = 16.4%, *p*-value < 0.001). The second regression model, which added the interaction term (TikTok addiction score ∗ sex), explained an additional 0.7% of the variance (ΔR^2^ = 0.7%, *p*-value = 0.003), bringing the total variance explained to 17.1% (R^2^ = 17.1%, *p*-value < 0.001). In the first model, TikTok addiction score had a significant effect on self-esteem. We found a negative association between TikTok addiction score and self-esteem. In the second model, both TikTok addiction score and sex remained significant, and the interaction term also significantly predicted self-esteem. Thus, sex moderated the association between TikTok addiction score and self-esteem. Simple slopes analysis indicated a stronger association between TikTok addiction score and self-esteem among males (Figure 5).

### 3.7. Association Between Problematic TikTok Use and Self-Esteem with Age as the Moderator 

**Figure 6:**
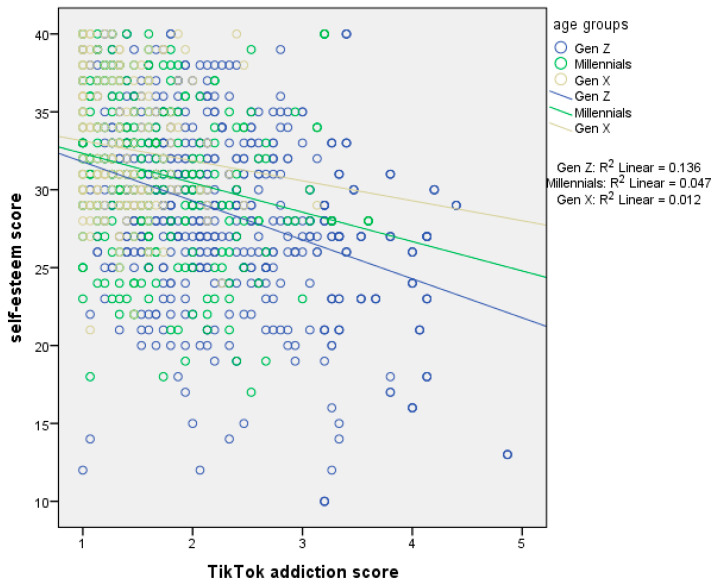
Simple slopes analysis with self-esteem score as the dependent variable and age as the moderator.

Table 7 presents moderation analysis with self-esteem score as the dependent variable and age as the moderator. The first regression model, which included the independent variable (centered TikTok addiction score) and the moderator (age), explained 18.4% of the variance in self-esteem score (R^2^ = 18.4%, *p*-value < 0.001). The second regression model, which added the interaction term (TikTok addiction score ∗ age), explained an additional 0.4% of the variance (ΔR^2^ = 0.4%, *p*-value = 0.024), bringing the total variance explained to 18.8% (R^2^ = 18.8%, *p*-value < 0.001). In the first model, TikTok addiction score had a significant effect on self-esteem. We found a negative association between TikTok addiction score and self-esteem. In the second model, both TikTok addiction score and age remained significant, and the interaction term also significantly predicted self-esteem. Thus, age moderated the association between TikTok addiction score and self-esteem. Simple slopes analysis indicated a stronger association between TikTok addiction score and self-esteem in Generation Z (Figure 6).

## 4. Discussion

TikTok daily use seems to have steady growth, largely among young generations. This immense spread underlines the major need for further investigation of TikTok’s reflection on individuals’ health status. Since the literature on this field is scarce, we examined the association between problematic TikTok use, procrastination, loneliness, and self-esteem.

Concerning daily use of TikTok, the sample had a mean time of use of 1.8 h. This finding aligns with other statistical sources concerning time on TikTok, which declare that the time users devote to TikTok may span from 52 to 150 min or more (Oberlo, 2023; Statista, 2025). Female and male participants spent almost the same time on TikTok daily, while, among generations, it was shown that first comes Generation Z, followed by the Millennials and, last, Generation X. These results agree with general statistics, supporting that two genders share almost equal time on TikTok, with Generation Z having the largest period daily (Oberlo, 2023; Statista, 2025). It is of great interest that the mean daily use of social media in this study was 3.3 h; since the mean for TikTok daily use was found to be 1.8 h, it means that the participants devote more than half of their social media time to TikTok. Although 92.9% of the sample had at least two more accounts on other social media, they preferred spending more time on TikTok. This finding verifies previous studies and statistics, which support that TikTok has prevailed over other social media, proving itself more interesting and addictive in contrast to other social media platforms (Wallaroomedia, 2024).

According to our results, there was a positive association between TikTok addiction score and procrastination score especially among females and Generation Z. These results indicate that, the more time is spent on TikTok, the more the users push their real-world responsibilities to the background of priorities. Generation Z is the one with the highest levels of TikTok use daily among all other generations. Furthermore, it is supported that women tend to procrastinate more than men due to a variety of reasons, such as stress, ideal standards of perfection, etc. (Balkis & Duru, 2024; Flett et al., 2016; Niermann & Scheres, 2014). As the literature supports, procrastination occurs either by failing to self-regulate or because of fear of missing out. Either way, users ignore their responsibilities and prioritize their time on TikTok and social media (Bao & Li, 2025; X. Li & Ye, 2022). The literature has underlined that procrastination associated with social media addiction frequently leads to poor sleep (Hale & Guan, 2015) and poor academic and school performance (Chao et al., 2023; Naushad et al., 2025; Zhou et al., 2024; Rasouli et al., 2025).

Another finding was that our sample indicated a positive association between TikTok addiction score and loneliness score. This association was more prominent among males and Generation X. Generation X finds TikTok addictive, ending up “absorbed” (Statista, 2023). Both social media and TikTok, according to other studies, have been associated with negative consequences for users, leading to poor mental health, such as loneliness, depression, stress, and poor sleep (Conte et al., 2025; Muñoz-Rodríguez et al., 2023; Harries et al., 2025; Chao et al., 2023). According to other studies, individuals, and most frequently boys, are feeling isolated when they go offline and especially when they are away from social media (including TikTok), declaring lack of communication and a feeling of isolation (Conte et al., 2025; Muñoz-Rodríguez et al., 2023; Savoia et al., 2021; Sipal et al., 2011). This feeling is attributed to the “fear of missing out” phenomenon, where individuals fear that they are missing important information and social updates when they go offline (Bao & Li, 2025; X. Li & Ye, 2022; Wu et al., 2025). Yet, this absorbance in the digital world costs a great load of real-world communication and interaction.

Moreover, our results indicated a negative association between TikTok addiction and self-esteem. This association was more prominent among males and Generation Z. This finding comes to be added to in the general context that TikTok affects mental health negatively, including self-esteem. More specifically, it is supported that prolonged TikTok use leads users to immerse themselves in a digital world where unrealistic body and lifestyle standards dominate. This daily interaction with non-achievable standards and stereotypes drives them to self-esteem issues, body dissatisfaction, and damaged self-perception (Calogero et al., 2011). Self-esteem issues due to addictive use of TikTok have also derived from Conte et al.’s systematic review where TikTok has been appointed as a risk factor for poor mental health, body-image issues, and low self-esteem (Conte et al., 2025). Both our study and previous studies in the literature support that younger ages are more vulnerable to self-esteem issues, especially when it comes to TikTok (Statista, 2023; Chung, 2018; Bleidorn et al., 2016). Concerning self-esteem and gender, the literature is divided into two parts. Generally, women seem to be more burdened with self-esteem issues due to social and mental-related reasons; yet there are studies supporting that men suffer too from the social stereotypes, leading to low self-esteem (Bleidorn et al., 2016; Casale, 2020; Jouini et al., 2018; Kling et al., 1999; J. Li et al., 2022; Mayo, 2016; Roberts, 1991).

Our study had several limitations. First, we used a convenience sample of TikTok users in Greece. Although we covered the sample size requirements, our sample cannot be representative of TikTok users. Moreover, sampling through TikTok inbox messages may raise self-selection bias. It is probable that participants that share significant characteristics may participate in our study. For instance, our study included mainly females (75.4%) and Generation Z participants (53.6%) and, thus, this sex and age imbalance may introduce selection bias. This selection bias limits generalizability of our findings. Future studies should include random samples to produce more representative results. Second, we conducted a cross-sectional study and, therefore, we cannot establish a causal relationship between problematic TikTok use, procrastination, loneliness, and self-esteem. Thus, we cannot be sure whether problematic TikTok use affects procrastination, loneliness, and self-esteem or whether these variables pre-exist and lead to increased problematic TikTok use. Longitudinal studies that explore the association between problematic TikTok use, procrastination, loneliness, and self-esteem could add significant information. Third, we used valid tools to measure problematic TikTok use, procrastination, loneliness, and self-esteem. However, our participants may compromise their answers due to social desirability bias. Therefore, information bias is probable in our study. Moreover, information bias may be introduced by the measurement of confounders. For instance, we measured socioeconomic status through a self-reported assessment. Fourth, we eliminated several confounders in our study, such as sex, age, educational level, socioeconomic status, TikTok use per day, social media use per day, and social media accounts. However, several other variables may introduce confounding in the association between problematic TikTok use, procrastination, loneliness, and self-esteem. In this context, scholars should eliminate more confounders in future studies. For instance, personality characteristics, family member relationships, and sleep patterns may be considered as potential confounders. Also, mental health issues, psychiatric history, and pre-existing conditions should be considered as potential confounders in future research. Moreover, we used four different length and valid scales to measure problematic TikTok use, procrastination, loneliness, and self-esteem in this study. Therefore, it was difficult to use more scales to measure our confounders since more questions may reduce participation rate. For instance, we measured socioeconomic status via a single self-report item and, thus, information bias is probably introduced in this study. Future studies could use more valid and extended tools to measure confounders such as socioeconomic status. Additionally, investigation of potential mediators in the association between problematic TikTok use, procrastination, loneliness, and self-esteem may further add significant knowledge on the impact of TikTok use. Finally, our scales showed very good Cronbach’s alpha, but they are still self-report scales and, thus, information bias is probable. We should mention that Cronbach’s alpha for one subscale of the TTAS was 0.689, slightly lower than the value of 0.7. Scholars should obtain in the future more valid measurements of variables such as TikTok use, procrastination, loneliness, and self-esteem to reduce information bias.

## 5. Conclusions

This study aimed to enlighten and contribute to further understanding of TikTok and its consequences. Interventions should be taken into consideration, in order to reduce the negative effect of problematic TikTok use on mental health. Problematic TikTok use is a modifiable risk factor for poor mental health outcomes. In this context, skills-based micro-interventions, screen-time reduction with sleep safeguards, digital literacy with active mediation, and platform measures may improve intentional use and elevate high-quality content (Herriman et al., 2025). Policy makers, stakeholders, and healthcare professionals can mount an effective, ethical, and scalable response by combining these elements in programs with rigorous monitoring and continuous improvement. Additionally, there is a need for digital/media literacy with active parental/educator mediation for youth and platform features that nudge breaks, elevate evidence-based content, and provide transparent well-being tools (Plackett et al., 2023).

At the individual level, to deal with problematic TikTok use, users can use built-in screen time limits and schedule specific times for app use rather than scrolling impulsively. Also, they can incorporate alternative activities like exercise, hobbies, or socializing offline to replace excessive screen time (Pieh et al., 2025). If TikTok use interferes with sleep, users can enable “do not disturb” mode and avoid the app at least an hour before bedtime. For persistent difficulties, users should consider behavioral strategies such as cognitive restructuring or seek professional support. Finally, users can curate their feed by unfollowing harmful content and engaging with positive, evidence-based creators to make their experience healthier and more intentional (Radtke et al., 2021).

The present study faced a number of limitations, such as the cross-sectional nature of data, and, thus, the findings should be examined under strict scrutiny. Further investigations should be carried out, such as longitudinal studies, to reduce bias and improve knowledge and research in this field.

## Figures and Tables

**Table 1:** Demographic characteristics of our participants (N = 1033).

Characteristics	N	%	95% Confidence Interval
Sex			
Females	779	75.4	72.7 to 78.0
Males	254	24.6	21.9 to 27.3
Age ^a^	31.1	12.4	30.4 to 31.9
Age categories			
Generation Z	554	53.6	50.5 to 56.7
Millennials	295	28.6	25.8 to 31.4
Generation X	184	17.8	15.5 to 20.3
Educational level			
High school	409	39.6	36.6 to 42.7
University degree	373	36.1	33.2 to 39.1
MSc diploma	229	22.2	19.7 to 24.8
PhD diploma	22	2.1	1.3 to 3.2
Socioeconomic status ^a^	6.2	1.5	6.1 to 6.3

^a^ mean, standard deviation.

**Table 2:** Moderation analysis (linear regression models) with procrastination score as the dependent variable and sex as the moderator.

	Unstandardized Coefficient Beta	95% CI for Beta	Standardized Coefficient Beta	*p*-Value	Adjusted R^2^ (%)
**Model 1**					32.1
TikTok addiction score	5.206	4.743 to 5.660	0.566	<0.001	
Sex	0.739	0.119 to 1.597	0.043	0.014	
**Model 2**					32.5
TikTok addiction score	5.560	5.019 to 6.100	0.604	<0.001	
Sex	3.331	1.108 to 5.553	0.195	0.003	
Interaction TikTok addiction score ∗ sex	−1.317	−2.360 to −0.275	−0.170	0.013	

CI: confidence interval.

**Table 3:** Moderation analysis (linear regression models) with procrastination score as the dependent variable and age as the moderator.

	Unstandardized Coefficient Beta	95% CI for Beta	Standardized Coefficient Beta	*p*-Value	Adjusted R^2^ (%)
**Model 1**					36.2
TikTok addiction score	5.596	5.069 to 6.124	0.591	<0.001	
Age	−0.183	−0.219 to −0.148	−0.300	<0.001	
**Model 2**					36.8
TikTok addiction score	5.208	4.583 to 5.834	0.550	<0.001	
Age	−0.039	−0.078 to −0.012	−0.063	0.041	
Interaction TikTok addiction score ∗ age	−0.061	−0.114 to −0.008	−0.069	0.024	

CI: confidence interval.

**Table 4:** Moderation analysis (linear regression models) with loneliness score as the dependent variable and sex as the moderator.

	Unstandardized Coefficient Beta	95% CI for Beta	Standardized Coefficient Beta	*p*-Value	Adjusted R^2^ (%)
**Model 1**					16.9
TikTok addiction score	0.940	0.813 to 1.066	0.413	<0.001	
Sex	−0.888	−1.256 to −0.243	−0.213	0.008	
**Model 2**					17.3
TikTok addiction score	0.845	0.697 to 0.992	0.371	<0.001	
Sex	−0.807	−1.415 to −0.199	−0.191	0.009	
Interaction TikTok addiction score ∗ sex	0.353	0.068 to 0.638	0.184	0.015	

CI: confidence interval.

**Table 5:** Moderation analysis (linear regression models) with loneliness score as the dependent variable and age as the moderator.

	Unstandardized Coefficient Beta	95% CI for Beta	Standardized Coefficient Beta	*p*-Value	Adjusted R^2^ (%)
**Model 1**					16.9
TikTok addiction score	0.921	0.776 to 1.065	0.405	<0.001	
Age	0.034	0.022 to 0.042	0.211	<0.001	
**Model 2**					18.2
TikTok addiction score	1.116	0.946 to 1.286	0.490	<0.001	
Age	0.024	0.012 to 0.046	0.189	<0.001	
Interaction TikTok addiction score ∗ age	0.031	0.016 to 0.045	0.145	<0.001	

CI: confidence interval.

**Table 6:** Moderation analysis (linear regression models) with self-esteem score as the dependent variable and sex as the moderator.

	Unstandardized Coefficient Beta	95% CI for Beta	Standardized Coefficient Beta	*p*-Value	Adjusted R^2^ (%)
**Model 1**					16.4
TikTok addiction score	−2.784	−3.168 to −2.400	−0.406	<0.001	
Sex	2.878	0.913 to 4.792	0.236	<0.001	
**Model 2**					17.1
TikTok addiction score	−2.436	−2.883 to −1.989	−0.355	<0.001	
Sex	2.724	0.885 to 4.563	0.214	0.004	
Interaction TikTok addiction score ∗ sex	−1.298	−2.160 to −0.435	0.003	0.015	

CI: confidence interval.

**Table 7:** Moderation analysis (linear regression models) with self-esteem score as the dependent variable and age as the moderator.

	Unstandardized Coefficient Beta	95% CI for Beta	Standardized Coefficient Beta	*p*-Value	Adjusted R^2^ (%)
**Model 1**					18.4
TikTok addiction score	−2.228	−2.660 to −1.796	−0.325	<0.001	
Age	0.074	0.046 to 0.102	0.168	<0.001	
**Model 2**					18.8
TikTok addiction score	−1.910	−2.422 to −1.398	−0.278	<0.001	
Age	0.094	0.061 to 0.127	0.213	<0.001	
Interaction TikTok addiction score ∗ age	0.050	0.007 to 0.094	0.079	0.024	

CI: confidence interval.

## Data Availability

The original data presented in this study are openly available in FigShare at doi.org/10.6084/m9.figshare.28903820.

## Abbreviations

The following abbreviations are used in this manuscript:

CFI	Comparative fit index
CI	Confidence interval
IPS	Irrational Procrastination Scale
RMSEA	Root mean square error of approximation
RSES	Rosenberg Self-Esteem Scale
TLI	Tucker–Lewis index
TTAS	TikTok Addiction Scale
UCLA-LS-3	UCLA 3-Item Loneliness Scale
